# Impact of Intergenerational *Shokuiku* (Food and Nutrition Education) Programs on Alleviating Loneliness in Japanese Communities across Ages

**DOI:** 10.3390/nu16111661

**Published:** 2024-05-28

**Authors:** Kayo Kurotani, Rin Katane, Momoko Nagashima, Miho Saegusa, Nonoka Yokode, Nakamichi Watanabe, Kazunori Ohkawara

**Affiliations:** 1 Graduate School of Life Sciences, Showa Women’s University, 1-7-57 Taishido, Setagaya-ku, Tokyo 154-8533, Japan; nakamich@swu.ac.jp; 2 Department of Health Sciences, Showa Women’s University, 1-7-57 Taishido, Setagaya-ku, Tokyo 154-8533, Japan; rinleon66@gmail.com (R.K.); momoo.univ@gmail.com (M.N.); miho.yasu.s@icloud.com (M.S.); xiangblue629@gmail.com (N.Y.); 3 Graduate School of Informatics and Engineering, University of Electro-Communications, 1-5-1 Chofugaoka, Chofu, Tokyo 182-8585, Japan; ohkawara_kazunori@e-one.uec.ac.jp

**Keywords:** intergenerational program, *Shokuiku*, loneliness, social capital, multigenerational exchange, Japanese

## Abstract

As loneliness is a risk factor for mental and physical health problems in various age groups, this study aimed to explore the impact of the intergenerational *Shokuiku* (food and nutrition education) program (IGSP) on loneliness in a Japanese community. This single-arm intervention study conducted between 2022 and 2023 included children (n = 21), guardians (n = 16), university students (n = 3), and older adults (n = 6). The IGSP was a one-day program that included participants making and eating their own bread, butter, and sorbet. Loneliness was measured using the Five-item Loneliness Scale for Children (Five-LSC; Japanese) and the three-item UCLA Loneliness Scale (Japanese; for adults) with other direct questions. Social capital, including civic participation, social cohesion, and reciprocity, was assessed using a self-administered questionnaire. The Five-LSC score significantly decreased post-intervention (*p* = 0.04). There was a significant increase in adults who reported not feeling lonely (*p* = 0.001). However, the UCLA Loneliness Scale scores did not show any significant changes. A positive change in social cohesion, including community contribution (*p* = 0.001) and attachment (*p* = 0.002), was observed among adults. This study suggests that IGSPs have a positive impact on loneliness in children and a partly positive one in adults. These findings emphasize the potential of intergenerational programs to reduce loneliness in communities.

## 1. Introduction

Loneliness is a risk factor for both mental and physical morbidity [1,2]. Moreover, it is related to physical and social frailty [3,4] and cognitive decline [5] among older adults, oral health [6] among adults, and a higher risk for suicidal behavior [7] and depression [8] among adolescents. A meta-analysis of 345 studies reported that loneliness levels increased linearly with increasing calendar years between 1976 and 2019 [9]. In addition, a meta-analysis across 113 countries indicated that problematic levels of loneliness are experienced by a substantial proportion of the population in many countries [10].

According to a global survey, the prevalence of feelings of loneliness among Japanese adults is relatively low [11]. However, a nationwide government survey in 2022 in Japan reported that 20.7% and 40.3% of people over the age of 16 years felt lonely at least sometimes and at least occasionally, respectively [12]. A survey conducted in Japan reported elevated levels of loneliness among young and middle-aged adults [12]. A study in the Netherlands showed a linear trend [13], a study in the UK showed a U-shaped pattern (peaking in early and late adulthood) [14], and a study in Germany showed a complex nonlinear trajectory with elevated loneliness levels among young adults and the oldest adults [15]. Thus, the findings on the association between age and loneliness have been inconsistent. Moreover, most interventions and observational studies focusing on loneliness have been conducted among older adults.

A meta-analysis of 11 random control trials and 5 observational studies found moderate evidence to support the conclusion that regular group-based sessions, usually 2–4 months in duration, were associated with reduced loneliness among older adults [16]. Additionally, the meta-analysis showed that regular group exercises may be associated with only small reductions in loneliness [16]. Furthermore, a recent randomized control trial showed a single-session mindfulness and compassion intervention did not reduce loneliness in young adults [17]. To the best of our knowledge, there is limited evidence for the effect of a single-session intervention on loneliness. Thus, the evidence remains insufficient to draw conclusions about the effectiveness of regular group-based activities and individual in-person interactions among older adults.

Previous studies have revealed the effects of intergenerational exchanges in older adults and children [18,19,20,21,22]. Moreover, studies have assessed its positive effects on social capital [23,24,25]. However, the evidence of intergenerational exchanges among children, young adults, middle-aged adults, and older adults is limited. 

In 2023, the Japanese government published the Act for Promotion of Policy for Loneliness and Isolation [26]. Based on this act, efforts are being made to address isolation and loneliness. The key priority of this Act is the establishment of places where individuals can feel a sense of community and experience meaningful “connections” with others. One avenue for establishing such places is the promotion of *Shokuiku* activities. In Japan, *Shokuiku* forms the foundation of human life and is integral to intellectual, moral, and physical education. It aims to educate individuals about food and nutrition, empowering them to make informed choices for their own well-being through diverse experiences. Children’s cafeterias, one of the *Shokuiku* activities, are places for multigenerational community and communication, as local children and adults are able to eat together [27]. Such *Shokuiku* activities are expected to address isolation and loneliness.

To address the gaps in the literature, this study examined the effect of a single-session intergenerational *Shokuiku* (food and nutrition education) intervention on loneliness in a Japanese community setting.

## 2. Materials and Methods

### 2.1. Study Design

This was a single-arm interventional study. We held a one-day intergenerational *Shokuiku* (food and nutrition education) program (IGSP) aimed at alleviating loneliness in December 2022, as well as in July and August 2023. Flyers were distributed at local children’s cafeterias and associated events to recruit participants for the study. The participants were primarily residents living in the vicinity of the Jindaiji Digital Living Lab in Chofu, Tokyo, and comprised a multigenerational population, including elementary school children aged 6–12 years, as of 2022, and their guardians. In addition, university students and older adults were included in this study in 2023. Written informed consent was obtained from all the participants. A self-report questionnaire was administered before and immediately after the event. This study was conducted in accordance with the Declaration of Helsinki and the Ethical Guidelines for Medical and Health Research Involving Human Subjects. The study protocol was approved by the Ethics Committee of Showa Women’s University (KH 23-10).

### 2.2. Study Population

In 2022, 12 children and 10 parents (middle-aged adults) participated in this study. In 2023, the participation was expanded to include 9 children, 3 university students (young adults), 6 middle-aged adults, and 6 older adults (aged 68–82 years). Therefore, a total of 21 children, 25 adults (3 young adults, 16 middle-aged adults, and 6 older adults) participated in this study.

### 2.3. The Intergenerational Shokuiku Program

The participants applied for a free bread-making workshop and participated in a one-day program. The IGSP comprised approximately eight participants from various age groups. The IGSP involves hands-on activities related to bread making, including shaping, fermentation, baking, and tasting. During bread fermentation, participants engaged in lecture sessions on bread-making as well as hands-on activities such as butter-making and sorbet-making. To enhance communication among participants during the hands-on activities, individuals from different generations were paired together. Additionally, during the tasting session, participants offered their favorite self-made bread to others, expecting fellow participants to comment on and engage in conversations about the bread. Staff members provided support to ensure lively conversations among the participants.

### 2.4. Measurement of Loneliness

Loneliness was measured using a self-administered questionnaire, administered before and immediately after the *Shokuiku* program for both children and adults. For children in 2023, loneliness was measured using the Five-item Loneliness Scale for Children (Five-LSC) in Japanese [28]. The five questions were as follows: Do you feel that you are alone? Do you think that there is no body to play with? Do you feel left behind by the people around you? Do you think that no one would help you if you were in trouble? Do you feel lonely? The responses were rated on a 4-point Likert scale ranging from 1 (strongly disagree) to 4 (strongly agree). The average score of the five items was computed, with higher scores indicating greater loneliness. The Five-item LSC scale was developed with reference to the definition of loneliness in childhood and adolescence by Parkhurst and Hopmeyer [29]. According to this definition, loneliness is characterized by emotional tendencies such as feeling ‘sad’ and ‘painful’, and is often triggered by recognizing oneself as alone or lonely, or by experiencing a lack of relationships and intimacy with others. The Five-LSC scale has been shown to have good reliability and validity [28]. Among adults, the question about loneliness was asked directly: how often do you feel lonely? The response categories were never, rarely, occasionally, sometimes, often, and always. Loneliness among adults was also measured using the three-item University of California, Los Angeles (UCLA) Loneliness Scale [30] in Japanese [31,32]. The three questions were as follows: How often do you feel that you lack companionship? How often do you feel left out? How often do you feel isolated from others? The responses were coded as 1 (never), 2 (rarely), 3 (sometimes), or 4 (always). The scores of the three questions were summed, with higher scores indicating greater loneliness (ranging from 3 to 12). The three-item loneliness scale has a satisfactory reliability and concurrent and discriminant validity [30,31,32].

### 2.5. Measurement of Social Capital

Social capital, including civic participation, social cohesion, and reciprocity, was assessed using a self-administered questionnaire for adults, adapted from questions developed by Saito et al. [33]. Among the social capital items, social cohesion, including community trust, norms of reciprocity, and community attachment, were measured before and immediately after the IGSP. Civic participation and reciprocity were measured either before or immediately after the program because we did not expect these aspects to be changed by the IGSP. 

### 2.6. Other Factors

Demographic and lifestyle factors were assessed using self-administered questionnaires. Information on sex, age (school age in children), breakfast consumption, and eating well-balanced meals was collected from both children and adults. The number of well-balanced meals, including staple dishes, main dishes, and vegetable dishes, was also assessed. For adults, we asked about their place of residence and the duration of their residency. After the IGSP, participants were asked whether they had made new acquaintances or friends, engaged in intergenerational interactions, or enjoyed the program.

### 2.7. Statistical Analysis

The analysis was conducted separately for children and adults because of differences in the measurement scales of loneliness. Changes in the loneliness scale were assessed using a paired *t*-test, and changes in the proportion of loneliness status were examined using Fisher’s exact test. We repeated the analysis after excluding individuals who were not living in the vicinity of the Jindaiji Digital Living Lab (the intervention venue). All statistical analyses were performed using the STATA software (version 17.0; StataCorp LLC, College Station, TX, USA). The statistical significance was set to *p* < 0.05. 

## 3. Results

The children’s characteristics are presented in Table 1. There was a high proportion of lower-grade school children (grade 1–4), girls, and those who had experience in bread-making, a liking for bread, and had breakfast almost daily. 

The adult participants’ characteristics are shown in Table 2. Mean (SD) age was 48.1 (17.6) years, ranging from 20 to 84 years. There was a high proportion of middle-aged adults, women, non-smokers, and those who had breakfast almost daily. Four adults were not residents living in the vicinity of the study venue.

Individual social capital, including civic participation and reciprocity among adults, is shown in Table 3. The participation rates in volunteer groups, sports groups, hobby activities, and study or cultural groups were 44.0%, 48.0%, 40.0%, and 32.0%, respectively. Additionally, more than 95% of the participants reported either receiving or providing emotional support or instrumental support.

The changes in loneliness among the children following the intervention are presented in Table 4. The Five-LSC scale score significantly decreased after the IGSP (*p* = 0.04). However, individual responses did not show statistically significant changes.

The proportion of adults who reported never feeling lonely significantly increased from 12.5% before to 20.8% after participating in the IGSP program (*p* = 0.001) (Table 5). However, no significant decrease was observed using the UCLA Loneliness Scale. While the percentage of individuals who always felt a lack of companionship decreased from 12.5% to 0.0% (*p* = 0.009), the proportion of those who sometimes felt left out or isolated from others showed a statistically significant increase (*p* < 0.001 and *p* = 0.004) after the program. The associations did not change even after excluding individuals who did not live in the study area (n = 4).

Changes in social cohesion were assessed only among adults (Table 6). Community contributions and attachment showed statistically significant positive changes (*p* = 0.001 and *p* = 0.002, respectively). The proportion of individuals who disagreed with community contributions decreased, whereas the proportion of those who were very attached to their communities increased. 

After excluding individuals not living in the study area (n = 4), both community contribution and attachment continued to show statistically significant positive changes (*p* = 0.01 and *p* = 0.008, respectively). Furthermore, community trust increased significantly (*p* = 0.048).

Among the participants, 70% of the adults and 43% of the children were able to make acquaintances or friends following the IGSP; all children and adult participants responded that they enjoyed the IGSP. Among the activities performed during the IGSP, the proportions of those who enjoyed butter-making (children: 88.9%; adults: 93.3%), sorbet-making (children: 77.8%; adults: 80.0%), eating self-baked bread (children: 77.8%; adult: 73.3%), and intergenerational exchange (children: 55.6%; adults: 93.3%) were high.

## 4. Discussion

To the best of our knowledge, this is the first study to examine the effects of the IGSP on loneliness among children and adults in Japan. The present study identified that loneliness in children decreased after the IGSP. While the effect on the UCLA loneliness scores were not identified, the proportion of adults who felt lonely decreased after the IGSP. Additionally, social capital, including community contribution and attachment, improved after the IGSP. We found a positive effect of the IGSP on loneliness in children and a partly positive one in adults. 

To date, intergenerational programs (IGP) aimed at facilitating cooperation and exchange among different age groups have been conducted worldwide. A systematic review of IGP among school-age children and older people [20] showed that two of the six studies identified the benefits of IGPs for children [34,35], while four of the seven studies identified the benefits of IGPs for older adults [35,36,37,38]. The systematic review interpreted the results as indicating that the meaningfulness of activities is essential for the beneficial effects of IGP, particularly the sense of usefulness [20]. Additionally, the feeling of being useful and competent may have brought enjoyment to both groups of participants [20]. Our results showed that all our participants, both children and adults, enjoyed the IGSP. Furthermore, we found a positive change in community contribution, suggesting that the IGSP activities were considered meaningful and useful.

The present study showed that the proportion of adults who felt lonely decreased after the IGSP; however, the UCLA loneliness scores did not change. Among the three items on the UCLA Loneliness Scale, the proportion of those who felt left out or isolated from others increased, although the proportion of those who felt a lack of companionship decreased. This suggests that our program might promote mutual exchange but not mutual understanding and acceptance. One possible reason for this may be the short duration of the program. The present IGSP was a one-day program, and the participants did not know each other before the program. Most previous studies have shown that participants met for approximately 6 months during IGPs [20]. Hence, several opportunities may be needed to construct confidential relationships among participants during the program. 

In the present study, activities in which participants cooperated with each other, such as making butter and sorbets, were popular among both children and adults. Additionally, we found that almost all adults enjoyed intergenerational exchanges. In the butter-making process, multigenerational participants cheered at each other and shook the fresh cream container together, indicating that cooperation among participants may promote intergenerational exchange.

This study demonstrates some strengths, including collection of data from a wide range of generations, reflecting the perspectives of youth and older adult participants. Moreover, it used programming that involved authority figures (e.g., guardians), which contributed to positive outcomes. This study had several limitations. First, the sample size was small, and we could not examine the effects of IGSP by generation. Second, loneliness was measured using self-report questionnaires, although we used validated loneliness scales for both children and adults [28,32]. In the present study, none of the children reported feeling lonely as assessed by the components of the Five-item LSC scale. Nevertheless, we observed a notable reduction in the overall Five-item LSC scale scores following the implementation of the IGSP, suggesting a discernible decrease in perceived loneliness levels among the participants. It is noteworthy that the Five-item LSC scale, employed in our assessment, aligns with the comprehensive definition of loneliness in childhood and adolescence proposed by Parkhurst and Hopmeyer [29]. Third, the program was a short one-day program that could only reveal the immediate effects of IGSP on loneliness; thus, the long-term effects may differ. This very short-term pre-post analysis may not evaluate the lasting impacts or capture the delayed effects on loneliness. The initial positive responses might not reflect genuine long-term changes. Therefore, a long-term pre-post analysis is necessary for future research. Fourth, selection bias may have affected the results because of the absence of a control group. Therefore, subsequent research should establish a control group and conduct a randomized trial. Finally, the generalizability of this study is limited due to subject bias. As opposed to a nationwide government survey on loneliness and isolation in Japan, which reported that 40.3% of people felt lonely, at least occasionally [12], only 12.5% of adults in our study reported feeling lonely at least occasionally. Further research is required to clarify the effects of intergenerational interventions on loneliness in other populations, with a larger sample.

## 5. Conclusions

This study suggests that the IGSP has a positive impact on loneliness in children and a partly positive one in adults in Japan. These findings emphasize the potential of such programs to promote social cohesion and reduce loneliness in communities.

## Figures and Tables

**Table 1:** Participant characteristics (children).

	n	(%)
**School age**		
Grade 1–2	10	(47.6)
Grade 3–4	7	(33.3)
Grade 5–6	4	(19.0)
**Sex**		
Boys	7	(33.3)
Girls	14	(66.7)
**Participation year**		
2022	12	(57.1)
2023	9	(42.9)
**Experience in bread making**		
Yes	15	(71.4)
No	6	(28.6)
**Liking for bread**		
Yes	20	(95.2)
No	0	(0.0)
Neutral	1	(4.8)
**Household chores ^1^**		
Everyday	0	(0.0)
Sometimes	18	(94.7)
Never	1	(5.3)
**Eating breakfast ^1^**		
Almost everyday	19	(100.0)
Fewer than 5 days/week	0	(0.0)
**Number of well-balanced meals ^1,2^**		
None	2	(10.5)
1	4	(21.1)
2	10	(52.6)
3	3	(15.8)

^1^ Number of respondents was 19. ^2^ Well-balanced meals were defined as a meal including staple dishes, main dishes, and vegetable dishes.

**Table 2:** Participant characteristics (adults).

	n	(%)
Age (years)	48.1 (mean)	17.6 (SD)
**Generation**		
Young adults	3	(12.0)
Middle-aged adults	16	(64.0)
Older adults	6	(24.0)
**Sex**		
Men	3	(12.0)
Women	22	(88.0)
**Participation year**		
2022	10	(40.0)
2023	15	(60.0)
**Residential area**		
Chofu	21	(84.0)
Other areas	4	(16.0)
**Duration of residency (mean (SD))**		
Chofu	21	(16.6)
Other areas	14.3	(8.0)
**Eating breakfast ^1^**		
Almost everyday	18	(75.0)
Fewer than 5 days/week	6	(25.0)
**Number of well-balanced meals ^1,2^**		
None	3	(12.0)
1	8	(32.0)
2	8	(32.0)
3	6	(24.0)
**Alcohol drinking status**		
Almost never	10	(40.0)
Less than 1–2 times/week	9	(36.0)
More than 3 times/week	6	(24.0)
**Smoking status**		
Non-smoker	23	(92.0)
Current smoker	2	(8.0)

^1^ Number of respondents was 24. ^2^ Well-balanced meals were defined as a meal including staple dishes, main dishes, and vegetable dishes.

**Table 3:** Civic participation and reciprocity among adults.

	n	(%)
**Civic participation**		
**Volunteer group**		
Yes	11	(44.0)
No	14	(56.0)
**Sports group**		
Yes	12	(48.0)
No	13	(52.0)
**Hobby activity**		
Yes	10	(40.0)
No	15	(60.0)
**Study or cultural group**		
Yes	8	(32.0)
No	17	(68.0)
**Skill teaching**		
Yes	2	(8.0)
No	23	(92.0)
**Reciprocity**		
**Received emotional support ^1^**		
Yes	23	(95.8)
No	1	(4.2)
**Provided emotional support ^1^**		
Yes	23	(95.8)
No	1	(4.2)
**Received instrumental support ^1^**		
Yes	24	(100.0)
No	0	(0.0)

^1^ Number of respondents was 24.

**Table 4:** Change in loneliness among children ^1^.

	Pre-Intervention		Post-Intervention		*p* Value ^2^
	n	(%)	n	(%)	
**I feel lonely ^3^**					
Strongly disagree	6	(66.7)	8	(88.9)	0.33
Disagree	3	(33.3)	1	(11.1)	
Agree	0	(0.0)	0	(0.0)	
Strongly agree	0	(0.0)	0	(0.0)	
**I feel that I am alone ^3^**					
Strongly disagree	5	(55.6)	8	(88.9)	0.44
Disagree	3	(33.3)	1	(11.1)	
Agree	1	(11.1)	0	(0.0)	
Strongly agree	0	(0.0)	0	(0.0)	
**I feel that I am left behind by others ^3^**					
Strongly disagree	5	(55.6)	7	(77.8)	0.17
Disagree	4	(44.4)	1	(11.1)	
Agree	0	(0.0)	1	(11.1)	
Strongly agree	0	(0.0)	0	(0.0)	
**I think that nobody helps me if I am in trouble ^3^**					
Strongly disagree	6	(66.7)	9	(100.0)	NA
Disagree	2	(22.2)	0	(0.0)	
Agree	1	(11.1)	0	(0.0)	
Strongly agree	0	(0.0)	0	(0.0)	
**I think that there is nobody to play with ^3^**					
Strongly disagree	7	(77.8)	8	(88.9)	0.22
Disagree	2	(22.2)	1	(11.1)	
Agree	0	(0.0)	0	(0.0)	
Strongly agree	0	(0.0)	0	(0.0)	
**Five-LSC scale (mean (SD))**	1.40	(0.46)	1.13	(0.28)	0.04

^1^ The data on loneliness among children, both pre- and post-intervention, was collected solely in 2023. ^2^ Fisher’s exact test was used for categorical variables and paired *t*-test was used for continuous variables. ^3^ The items of Five-LSC scale.

**Table 5:** Change in loneliness among adults.

	Pre-Intervention		Post-Intervention		*p* Value ^1^
	n	(%)	n	(%)	
**I feel lonely (direct)**					
Never	3	(12.5)	5	(20.8)	0.001
Rarely	18	(75.0)	16	(66.7)	
Occasionally	1	(4.2)	2	(8.3)	
Sometimes	2	(8.3)	1	(4.2)	
Always	0	(0.0)	0	(0.0)	
**I feel that I lack companionship ^2^**					
Never	6	(25.0)	6	(25.0)	0.009
Rarely	11	(45.8)	13	(54.2)	
Sometimes	4	(16.7)	5	(20.8)	
Always	3	(12.5)	0	(0.0)	
**I feel left out ^2^**					
Never	6	(25.0)	5	(20.8)	<0.001
Rarely	16	(66.7)	16	(66.7)	
Sometimes	2	(8.3)	3	(12.5)	
Always	0	(0.0)	0	(0.0)	
**I feel isolated from others ^2^**					
Never	5	(20.8)	5	(20.8)	0.004
Rarely	17	(70.8)	16	(66.7)	
Sometimes	2	(8.3)	3	(12.5)	
Always	0	(0.0)	0	(0.0)	
**UCLA Loneliness Scale**	5.88	(1.85)	5.79	(1.74)	0.82

^1^ Fisher’s exact test was used for categorical variables and paired *t*-test was used for continuous variables. ^2^ The items of three-item UCLA Loneliness Scale.

**Table 6:** Change in social cohesion among adults.

	Pre-Intervention		Post-Intervention		*p* Value ^1^
	n	(%)	n	(%)	
**Community trust**					
Trust very much	5	(20.0)	3	(12.0)	0.31
Moderately trust	19	(76.0)	21	(84.0)	
Neutral	1	(4.0)	1	(4.0)	
Do not trust	0	(0.0)	0	(0.0)	
Never trust	0	(0.0)	0	(0.0)	
**Community contribution**					
Agree very much	2	(8.3)	2	(8.3)	0.001
Moderately agree	17	(70.8)	17	(70.8)	
Neutral	5	(20.8)	6	(25.0)	
Do not agree	1	(4.2)	0	(0.0)	
Never agree	0	(0.0)	0	(0.0)	
**Community attachment**					
Very attached	4	(16.0)	5	(20.0)	0.002
Moderately attached	20	(80.0)	19	(76.0)	
Neutral	1	(4.0)	1	(4.0)	
Not attached	0	(0.0)	0	(0.0)	
Never attached	0	(0.0)	0	(0.0)	

^1^ Fisher’s exact test was used.

## Data Availability

The raw data supporting the conclusions of this article will be made available by the authors on request.
